# Histocompatibility Leukocyte Antigen-A29-Associated Retinal Vasculitis without Choroidal Lesions: A Report of 4 Cases

**DOI:** 10.3390/jcm12227023

**Published:** 2023-11-10

**Authors:** Estefanía Ramírez-Márquez, Sofía C. Ayala Rodríguez, Alejandra Santiago, Edgar De Jesus, Guillermo A. Requejo Figueroa, Mona M. Riskalla, Meghan E. Ryan, Dara Koozekanani, Armando L. Oliver

**Affiliations:** 1 Department of Ophthalmology, School of Medicine, Medical Sciences Campus, University of Puerto Rico, San Juan, PR 00936, USA; estefania.ramirez@upr.edu (E.R.-M.); sofiacayalarodriguez@gmail.com (S.C.A.R.); alejandra.santiago9@upr.edu (A.S.); edgar.dejesus1@upr.edu (E.D.J.); guillermo.requejo1@upr.edu (G.A.R.F.); 2 Division of Pediatric Rheumatology, Allergy, and Immunology, Department of Pediatrics, University of Minnesota Medical School Twin Cities, Minneapolis, MN 55455, USA; riskalla@umn.edu (M.M.R.); meghanr@umn.edu (M.E.R.); 3 Department of Ophthalmology and Visual Neurosciences, University of Minnesota Medical School Twin Cities, Minneapolis, MN 55455, USA; dkoozeka@umn.edu

**Keywords:** birdshot retinochoroidopathy, HLA-A29, retinal vasculitis

## Abstract

This study describes a cohort of patients presenting with histocompatibility leukocyte antigen (HLA)-A29-associated retinal vasculitis without choroidal lesions that may share clinical features with birdshot retinochoroiditis. The methods include a retrospective chart review of patients presenting with HLA-A29-associated retinal vasculitis without choroidal lesions. The data on the patients were entered retrospectively into a new database and analyzed. Four patients who had HLA-A29-associated retinal vasculitis without choroidal lesions were identified. The median age at presentation was 40 years (range: 14–71); 75% were female. At presentation, all four patients had a visual acuity of 20/50 or better in both eyes. All the eyes had mild vitritis, three eyes (37.5%) had cystoid macular edema, and two eyes (25%) had optic disc edema. All the patients required treatment with systemic steroids and immunosuppressive therapy. HLA-A29-associated retinal vasculitis without choroidal lesions appears to share many clinical features with birdshot chorioretinitis, including the need for systemic immunosuppressive therapy. Whether this entity represents an early form of birdshot retinochoroiditis or a more localized variant of the disease is a topic for additional studies.

## 1. Introduction

Birdshot retinochoroidopathy (BCR) is a chronic, usually bilateral, autoimmune posterior uveitis that is identified in 6% to 7.9% of patients with posterior uveitis [1]. This disease is characterized by cream-colored dots scattered diffusely through the fundus, mostly around the optic disc, and radiating out toward the midperiphery [2]. BCR is correlated with the histocompatibility leukocyte antigen (HLA)-A29 class I type [2,3]. This antigen has been reported in 80% to 100% of BCR patients and in 7% of the general population [2,3,4].

The current diagnostic criteria for BCR do not include HLA-A29-positive vasculitis in the absence of visual choroidal lesions on the examination of the fundus [2]. Ryan and Maumenee first described the typical BCR lesion in 1980 with the use of fundus examination and fluorescein angiography (FA) [2,5]. A recent study about the positive predictive value of FA patterns of vascular leakage in patients with BCR revealed that this disease is more likely to have a perineural retinal vascular leakage pattern [6]. Currently, the use of modern diagnostic tools, such as indocyanine green angiography (ICGA), allows for a new characterization of BCR through the visualization and monitoring of the presence of hypocyanescent dark dots and the fuzziness of choroidal vessels [2,7].

Since its initial description in the late 1970s, the treatment paradigm for BCR has undergone significant evolution [7,8]. Initially, the treatment focused on addressing cystoid macular edema using corticosteroids [7,8,9]. Later research, however, revealed that corticosteroid monotherapy in BCR often resulted in recurring cystoid macular edema and worsening outer retinal dysfunction, leading to peripheral visual field loss [7,8,9]. This degradation was observed even when inflammation and cystoid macular edema appeared absent clinically, highlighting the limitations of corticosteroid monotherapy in preventing disease progression [7,8]. As a result, the emphasis has now shifted to chronic treatment strategies that incorporate immunosuppressive medications as alternatives to steroids [7,8,9,10]. These immunosuppressants have notably proven effective in both preventing cystoid macular edema and reversing visual field loss due to outer retinal damage [7,8,9]. Typically, alongside systemic or local corticosteroids, antimetabolic agents such as mycophenolate mofetil and methotrexate are prescribed upon the diagnosis. Infliximab and adalimumab may serve as secondary immunomodulatory choices when an optimal steroid-sparing effect is not initially achieved [7,8,9,10]. For patients resistant to both anti-metabolites and anti-TNF alpha inhibitors, anti-interleukin-6 agents might be a viable option [10].

BCR is a potentially blinding disease in which patients benefit from the combined use of steroids or immunosuppressive pharmacotherapy [8,9,10,11]. It has been proposed that early treatment may prevent the appearance of BCR lesions [2,8,9,10,11]. We herein describe a cohort of patients presenting with HLA-A29-associated retinal vasculitis without choroidal lesions that may share clinical features with birdshot retinochoroiditis.

## 2. Materials and Methods

Medical records spanning from 2005 through 2021 were obtained from the databases of two private practices in Puerto Rico, as well as the University of Minnesota Department of Ophthalmology. The study’s inclusion criteria focused on patients with a diagnosis of HLA-A29-associated retinal vasculitis, specifically excluding cases with classic BCR lesions present in fundus examination. A total of four patients met these criteria and were subsequently included in the comprehensive analysis.

The data derived from the retrospective review of medical records pertaining to HLA-A29-positive patients with associated retinal vasculitis in the absence of lesions on fundus examination were systematically entered into a newly established database to facilitate a comprehensive analysis. The dataset encompassed a range of demographic information, such as sex, date of birth, and age at the time of initial presentation. Additionally, this database incorporated a set of crucial metrics for each patient, acquired during the initial presentation, including visual acuity, intraocular pressure, results from Ishihara color plates testing, and the assessment of ocular manifestations, notably vitritis and cystoid macular edema. Vitritis, in this context, was specifically defined as the presence of vitreous cells discerned through a slit-lamp examination. All patients underwent intravenous fluorescein angiograms and ICGA.

The patients underwent an extensive systemic workup, which encompassed a series of diagnostic tests and evaluations tailored to each patient’s unique clinical presentation. The serologic studies included a fluorescent treponemal antibody absorption (FTA-ABS) test, a rapid plasma reagin (RPR) test, an assessment of lysozyme activity, an assay for West Nile virus, a Lyme antibodies test, a urinalysis, a comprehensive metabolic panel (CMP), a complete blood count (CBC), a test for hepatitis, HLA-B51 genotyping, tests for myeloperoxidase and proteinase 3 antibodies, an anti-dsDNA test, a C-reactive protein (CRP) test, an erythrocyte sedimentation rate (ESR) test, a test for Epstein–Barr virus (EBV), an EBV nuclear antigen (EBNA) antibody test, an antineutrophil cytoplasmic antibodies (ANCA) test, an antinuclear antibodies (ANA) test, an angiotensin-converting enzyme (ACE) test, and a QuantiFERON-Tb Gold Plus (QTF-Plus) test. In addition, the workup included a chest X-ray, a head computed tomography (CT) scan, brain magnetic resonance imaging (MRI), a bone scan, and a gallium scan, when clinically indicated. Descriptive analyses of both the ocular and systemic findings at the time of presentation were conducted to assess the data. The study was conducted in compliance with the principles outlined in the Declaration of Helsinki. The protocol received approval from the Internal Review Board of the University of Puerto Rico Medical Sciences Campus.

## 3. Results

### 3.1. Case 1

A 27-year-old female presented with a history of photopsia and floaters in both eyes (OU), for one year prior to her evaluation. Her past medical history was remarkable for Hashimoto thyroid disease. Her family history was remarkable for cancer in her maternal grandmother. The review of systems was remarkable for hypoglycemia.

**Figure 1:**
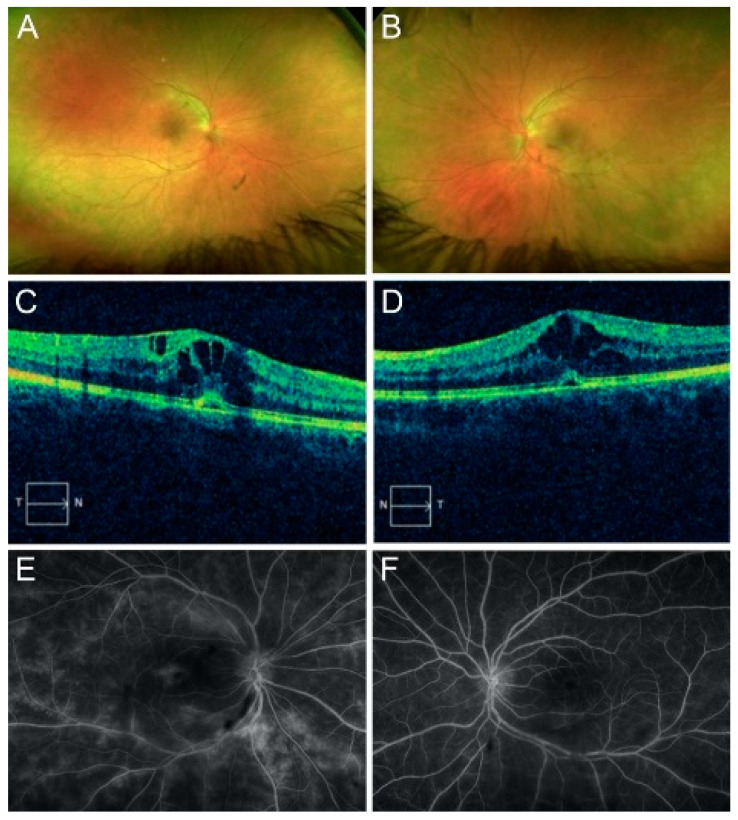
Fundus imaging at initial presentation of the case 1 patient. Right (**A**) and left (**B**) color photographs revealed the presence of vitreous cells and mild perivascular sheathing of the vessels in both eyes. Optical coherence tomography examination of the right (**C**) and left (**D**) macula cross-sections (temporal (T) to nasal (N) and N to T directions, as denoted by the arrow boxes, respectively) reveals cystoid macular edema with subretinal fluid. Right (**E**) and left (**F**) fluorescein angiograms show branching vasculitis and diffuse leakage in both eyes.

The initial best-corrected visual acuity (BCVA) was 20/30 in the right eye (OD) and 20/20 in the left eye (OS). The intraocular pressure was 15 mmHg OU. The confrontation visual field was full. A slit-lamp exam was unremarkable OU. An examination of the fundus revealed the presence of 3+ vitreous cells and mild sheathing of the vessels OU (Figure 1A,B). Optic disc edema and epiretinal membrane (ERM) were present OU.

Spectral-domain optical coherence tomography (OCT) revealed center-involving cystoid macular edema with a small subfoveal detachment OU (Figure 1C,D). FA revealed branching vasculitis and diffuse leakage OU (Figure 1E,F). ICGA revealed no hypocyanescent or hypercyanescent lesions OU. An electroretinogram revealed a normal scotopic response and an abnormal photopic response.

The patient was found to be HLA-A29 positive. A whole-body gallium scan revealed inflammation localized in the orbits. A bone scan revealed arthritic changes in the interphalangeal joint of the first finger of the left hand and the anterior aspect of her right ankle. The comprehensive workup, which included an FTA-ABS test, an RPR, a lysozyme analysis, a West Nile virus antibodies assay, a Lyme antibodies assay, a urinalysis, a chest X-ray, a CMP, a CBC, a hepatitis test, an ANA test, an ACE test, a QTF-Plus test, and a brain MRI, was unremarkable.

A diagnosis of HLA-A29-associated retinal vasculitis was made; the patient was started on prednisone (60 mg oral, once per day) and mycophenolate mofetil (1.5 g oral, twice per day). The patient only achieved partial improvement; therefore, adalimumab was added as an adjuvant therapy to aid in tapering the concomitant steroid therapy. After a gradual tapering of the prednisone and upon reaching a 20 mg dose, she developed cystoid macular edema, which was treated with an intravitreal dexamethasone implant in each eye, which caused significantly elevated intraocular pressure.

As the condition was refractory to mycophenolate mofetil and adalimumab as steroid-sparing agents, she was started on repository corticotropin injections (80 U, twice per week), which resulted in the resolution of the macular edema and significant improvement of the retinal vasculitis, leading to the subsequent tapering and discontinuation of the systemic steroids. Two years following treatment, she maintained a visual acuity of 20/25 and remained asymptomatic.

### 3.2. Case 2

A 43-year-old female was referred for the further evaluation of her flashes and retinal perivasculitis. Her past medical history included diabetes, hypercholesterolemia, and hypothyroidism. Her past social history and review of systems were unremarkable.

**Figure 2:**
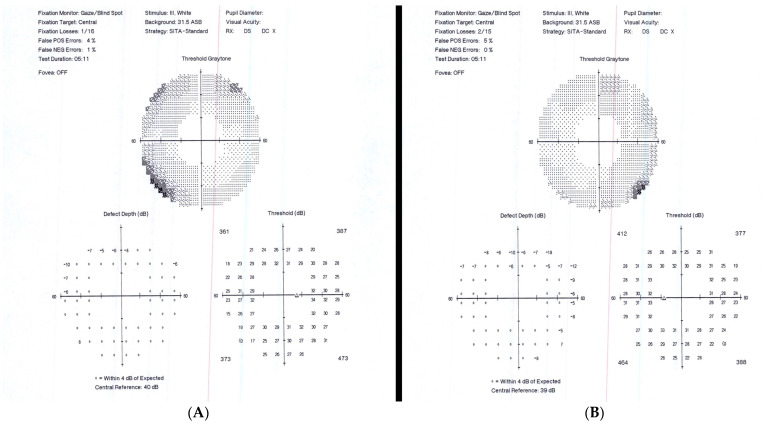
Bilateral visual field upon presentation (Humphrey, central 60-4 threshold test, stimulus III, white, Swedish interactive thresholding algorithm Fast) of case 2. Right (**A**) and left (**B**) visual fields revealed depressions at the superior nasal level and one inferior area of depression, as illustrated in the threshold grayscale, the defect depth, and threshold (dB), which further reveal the numerical values supporting the superior nasal level depressions and the single inferior area of depression.

Upon the initial ophthalmic exam, our patient had a BVCA of 20/20 OD and 20/20 OS. The intraocular pressure was 15 mmHg OU. A Humphrey visual field (60-4) test revealed peripheral constriction OU (Figure 2). A slit-lamp exam was remarkable for a stable eyelid hemangioma OS and was otherwise within normal limits. A fundus examination revealed predominant infratemporal sheathing of the vessels OU compatible with retinal vasculitis and 0.5+ cells in the vitreous. An ICGA revealed no hypocyanescent or hypercyanescent lesions OU, and a FA displayed a single hypercyanescent area temporal to the macula, with leakage that seemed to emerge from a single choroidal vessel.

The patient was HLA-A29 positive. A brain MRI was remarkable for a cyst in the cavum velum interpositum but was otherwise unremarkable. A comprehensive workup, which included an FTA-ABS test, an RPR test, a lysozyme analysis, a West Nile virus antibodies assay, a Lyme antibodies assay, a urinalysis, a chest X-ray, a CMP, a CBC, a hepatitis test, an ANA test, an ACE test, and a QTF-Plus test, was unremarkable.

A diagnosis of HLA-A29-associated retinal vasculitis was made, and the patient was started on prednisone (60 mg oral, daily) and mycophenolate mofetil (1.5 g oral, twice daily). Over the course of the following ten years, the patient required long-term therapy with mycophenolate mofetil and prednisone. During this period, she experienced several flare-ups when attempting to taper down both medications. During the course of therapy, she maintained 20/20 visual acuity. Additionally, during her follow-up over ten years, the patient underwent ICGA at the seventh, eighth, and ninth years. All of these ICGA examinations were negative for hypocyanescent spots, confirming the findings of her initial ICGA.

### 3.3. Case 3

A 14-year-old female presented with a history of floaters, blurred vision, and dyschromatopsia. She had no prior history of systemic disease. Her past social history and review of systems were unremarkable.

A comprehensive ophthalmic exam revealed a BVCA of 20/40 OD and 20/50 OS. The intraocular pressure was 14 mmHg OD and 15 mmHg OS. A slit-lamp exam was unremarkable OU. The fundus examination revealed vitritis in both OD (1 to 2+) and OS (2+). Fine cells, macular edema, and retinal vasculitis were present OU. An OCT scan revealed cystoid macular edema OU. An FA revealed macular and vascular leakage.

A comprehensive workup revealed her to be HLA-A29 positive. All tests, including lysozyme, chest X-ray, HLA-B51, myeloperoxidase and proteinase 3 antibodies, dsDNA, CRP, ESR, EBV, EBNA, ANCA, ACE, ANA, CMP, and QTF-Plus, were unremarkable.

The patient was initially treated with topical and oral steroids; nevertheless, her disease had progressed by the one-month follow-up examination. A pediatric rheumatologist started the patient on pulse intravenous steroids, mycophenolate (750 mg in the morning), and mycophenolate (1000 mg at night) in addition to the topical and oral prednisone.

At the follow-ups at months two through six, the patient experienced a stable resolution of her blurred vision, dyschromatopsia, and marked improvement in terms of her eye floaters. An examination revealed bilateral fine cells in the anterior chamber. The posterior chamber was remarkable for trace vitritis, the improvement of her retinal hemorrhage, and macular edema but no subretinal fluid.

Seven months after the patient’s initial visit, she presented with a new retinal hemorrhage OD. The new findings indicated deterioration of the disease in the OD and an improvement in the OS. For this reason, the patient’s therapy with prednisone was increased to 20 mg, twice daily, and cyclosporine (100 mg, twice daily) was added. She continued to take mycophenolate (750 mg) in the morning and mycophenolate (1000 mg) at night. A month later, the patient also received PRP laser treatment in both eyes.

Two months later, she had progressive bilateral improvement as evidenced by an improvement to her retinal hemorrhage, trace cystoid macular edema, and regressing retinal neovascularization. She remained on mycophenolate, cyclosporine, topical prednisone, and oral prednisone. She was started on adalimumab (40 mg subcutaneous every 14 days then after 4 months to every 7 days), which was well tolerated and allowed for the subsequent tapering and total discontinuation of the cyclosporine and systemic steroids.

### 3.4. Case 4

A 71-year-old male presented with floaters and flashes. His ocular history was remarkable for pseudophakia OU. He had no prior history of systemic disease. His past social history and review of systems were unremarkable.

A comprehensive ophthalmic exam revealed a BCVA of 20/30 OD and 20/20 OS. The intraocular pressure was 14 mmHg OU. An OCT scan confirmed the presence of vitreous cells and epiretinal membrane OU. An FA showed old venous sheathing OU but no evidence of active vasculitis. An ICGA revealed hypocyanescent lesions OU.

A comprehensive workup revealed him to be HLA-A29 positive. Further tests, including an FTA-ABS, an RPR, a chest X-ray, an ACE, and a QTF-Plus, were unremarkable.

The patient received treatment with mycophenolate mofetil, cyclosporine, prednisone, methotrexate, and infliximab, which resulted in adequate improvement.

## 4. Discussion

Our impression was that patients may present with HLA-A29-associated retinal vasculitis without the traditional birdshot retinochoroiditis choroidal lesions. Our results suggested that HLA-A29-associated retinal vasculitis might share clinical features (such as vitritis, cystoid macular edema, bilateral optic nerve swelling, and the need for aggressive systemic immunosuppressive therapy) with BCR. These characteristics led to the hypothesis that HLA-A29 retinal vasculitis may be a subtype of BCR that is limited to the retina and therefore does not present choroidal lesions.

An international consensus conference established the research criteria for the diagnosis of BCR in the year 2002. The criteria outlined in the report included bilateral disease, the presence of at least three peripapillary BCR lesions, low-grade anterior segment intraocular inflammation, and low vitreous inflammation as the required criteria. In addition to the required criteria, the report identified several supportive findings, including retinal vasculitis, HLA-A29 positivity, and cystoid macular edema. The exclusion criteria comprised the presence of keratic precipitates, posterior synechiae, and the presence of infectious, neoplastic, or other inflammatory diseases that can cause multifocal choroidal lesions [11].

Utilizing these guidelines, we proposed diagnostic criteria for HLA-A29 retinal vasculitis without concurrent choroidal lesions. This includes HLA-A29 positivity, bilateral disease, low-grade anterior segment inflammation, low-grade vitreous inflammatory reaction, and retinal vasculitis. Additional clinical features that can support this diagnosis may involve optic disk swelling and cystoid macular edema. Conversely, the diagnosis should be reconsidered if there is any presence of hypopigmented choroidal lesions (“birdshot lesions”) on the fundus examination, granulomatous keratic precipitates, posterior synechia, ischemic retinal vasculitis, or the presence of infectious, neoplastic, or other inflammatory diseases that can cause retinal vasculitis. A summary of these criteria is presented in Table 1.

BCR is characterized by multifocal choroidal lesions, retinal vasculitis, and macular edema [3,11]. It is important to recognize the characteristics of this disease because if no treatment is initiated, chronic retinal dysfunction may occur and visual field deterioration may progress with time, bringing with them significant potential for irreversible visual loss [3,12]. The duration of a given individual’s BCR disease prior to initiating therapy is a risk factor for that person’s loss of visual acuity; therefore, early treatment is vital to improving long-term outcomes [7,13].

Out of our four cases, all the patients received initial treatment with steroids and antimetabolic immunosuppressive agents. Furthermore, three of these cases required the addition of anti-TNF alpha inhibitors to achieve adequate disease control. For the patient in case 2, various follow-up ICGAs conducted over the subsequent 10-year period consistently revealed the absence of hypocyanescent spots, mirroring the findings from the initial ICGA. These findings suggest that early treatment may help prevent the development of classic BCR fundus lesions in these patients.

In the past, BCR was commonly treated with systemic steroid pharmacotherapy, which was initially a bridging therapy to steroid-sparing immunomodulatory therapy (IMT) [12]. However, the use of immunosuppressors may halt or improve visual field loss and prevent cystoid macular edema, an important cause of central visual acuity; therefore, all patients with BCR should be considered for IMT as soon as the diagnosis is established [8]. Additionally, several authors have stated that immunosuppressive treatment may even prevent the appearance of the traditional BCR fundus lesions [7,8,9,10,11,12,13,14]. These lesions are characterized by multiple ovoid, cream-colored spots that are scattered throughout the fundus but are mainly found around the optic disc and radiating out toward the mid-periphery [6,11].

**Figure 3:**
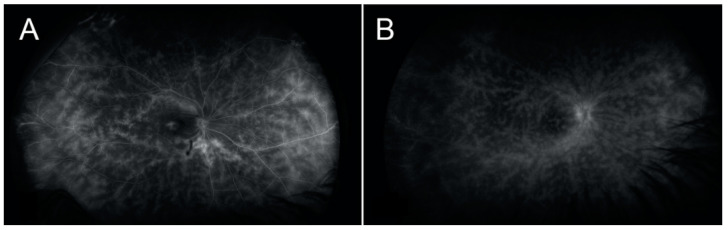
Photographs demonstrate side-by-side intravenous fluorescein angiograms of our case 1 patient (**A**) and a patient with classic birdshot (**B**). (**A**) The right eye shows diffuse branching vasculitis corresponding to areas of retinal vasculitis and leakage secondary to inflammation and active disease. (**B**) The traditional birdshot finding of retinal vasculitis.

ICGA is a useful diagnostic procedure to determine the extent of the inflammatory process (with choroidal involvement) in BCR [5]. The most characteristic ICGA findings involve hypofluorescent dark dots and the fuzziness of the choroidal vessels [5,6]. One of our patients presented with significant atrophic changes and hypofluorescent lesions, OD, very similar to what would be expected in a traditional case of BCR (Figure 3). Some authors have suggested that choroidal involvement may often be detected before the typical choroidal birdshot lesions are visible on fundoscopy and that early treatment may prevent the progressive development of these lesions [2,14,15,16]. However, of the four patients in our series who had had an ICGA performed, three did not show any choroidal lesions, suggesting the possibility that HLA-A29-associated posterior uveitis patients might suffer from different types of disease, with that of our patients being more localized to the retina than is that of the typical patient, in whom the choroidal lesions are clinically evident. It is also possible that our patients were diagnosed early in the course of the disease, and the subsequent early treatment prevented the development of the typical choroidal lesions [2].

The patients included in our study were classified as having HLA-A29-positive vasculitis because of the absence of visual choroidal lesions on the examination of the fundus. It may be that the patterns of vasculitis observed in our patients were similar to the BCR pattern (Figure 3) and that it was a coincidence that they were also HLA-A29 positive. Alternatively, it may be possible that BCR and HLA-A29-positive-associated vasculitis could represent similar entities or a different stage of the same disease; therefore, HLA-A29 genotyping should be considered in these cases. The identification of HLA-A29-positive-associated vasculitis may grant our patients the opportunity to prevent macular edema, visual field loss, and typical BCR lesions.

The study offers important insights into HLA-A29-associated retinal vasculitis in the absence of choroidal lesions. However, its retrospective design might introduce ascertainment and referral biases. Our sample size is relatively small, which could be attributed to the likelihood of undiagnosed cases. This may arise because HLA-A29 serology is not routinely checked in retinal vasculitis patients unless they exhibit the hypopigmented choroidal lesions typical of BCR. Future research should consider regular monitoring of these patients using visual field analysis and electroretinograms. It is also essential to evaluate the effectiveness of specific treatment algorithms, including antimetabolites, anti-TNF alpha inhibitors, and anti-interleukin-6 agents. A more exhaustive study of this clinical scenario is crucial to deepen our understanding further.

## 5. Conclusions

HLA-A29-associated retinal vasculitis without choroidal lesions displays clinical similarities to birdshot chorioretinitis, notably the necessity for systemic immunosuppressive treatment. Those with this specific type of retinal vasculitis seem to respond positively to therapies like antimetabolic agents and anti-TNF alpha inhibitors. It is advisable for physicians to include HLA-A29 genotyping in the diagnostic process for retinal vasculitis. Early detection of this patient subset can facilitate a more tailored treatment strategy. The suggested diagnostic criteria for this condition can assist clinicians in its timely identification and pave the way for future research in this area.

## Figures and Tables

**Table 1:** Proposed criteria for HLA-A29 retinal vasculitis without choroidal lesions.

Proposed Diagnostic Criteria for HLA-A29 Retinal Vasculitis without Choroidal Lesions	
Required characteristics	
	HLA-A29 positivity
	Bilateral disease
	Retinal vasculitis
	Low-grade anterior segment inflammation *
	Low-grade vitreous inflammatory reaction **
Supportive findings	
	Optic disk swelling
	Cystoid macular edema
Exclusion criteria	
	Presence of hypopigmented choroidal lesions (“birdshot lesions”) on the fundus examination
	Granulomatous keratic precipitates
	Posterior synechia
	Ischemic retinal vasculitis
	Presence of infectious, neoplastic, or other inflammatory diseases that can cause retinal vasculitis

* Defined as ≤1.0+ cells in the anterior chamber. ** Defined as ≤2.00+ vitreous haze.

## Data Availability

The data presented in this study are available on request to the corresponding author.
